# Impaired Expression of Type I and Type II Interferon Receptors in HCV-Associated Chronic Liver Disease and Liver Cirrhosis

**DOI:** 10.1371/journal.pone.0108616

**Published:** 2014-09-29

**Authors:** Partha K. Chandra, Feyza Gunduz, Sidhartha Hazari, Ramazan Kurt, Rajesh Panigrahi, Bret Poat, David Bruce, Ari J. Cohen, Humberto E. Behorquez, Ian Carmody, George Loss, Luis A. Balart, Tong Wu, Srikanta Dash

**Affiliations:** 1 Pathology and Laboratory Medicine, Tulane University Health Sciences Center, New Orleans, Louisiana, United States of America; 2 Department of Medicine, Gastroenterology and Hepatology, Tulane University Health Sciences Center, New Orleans, Louisiana, United States of America; 3 Transplant Surgery Section, Ochsner Medical Center, New Orleans, Louisiana, United States of America; Saint Louis University, United States of America

## Abstract

**Purpose:**

Chronic Hepatitis C Virus (HCV)-infected patients with liver cirrhosis (LC) respond poorly to interferon-alpha (IFN-α) and ribavirin (RBV) combination therapy, but the reason for this is unclear. We previously reported that HCV-infection induces endoplasmic reticulum (ER) stress and autophagy response that selectively down regulates the type I IFN-α receptor-1 (IFNAR1) and RBV transporters (CNT1 and ENT1), leading to IFN-α/RBV resistance. The goal of this study is to verify whether an increase in ER stress and autophagy response is also associated with the reduced expression of IFNAR1 and RBV transporters in chronic HCV-infected patients.

**Methods:**

Primary human hepatocytes (PHH) were infected with cell culture grown HCV particles (JFH-ΔV3-Rluc). HCV replication was confirmed by the detection of viral RNA by RT-qPCR and HCV-core protein by Western blotting. The ER stress and autophagy response and expression of IFN receptors and RBV transporters in HCV infected PHH and liver tissues derived from patients were measured by Western blotting.

**Result:**

HCV infection of PHH showed impaired expression of IFNAR1, IFNγR1 (Type II IFN receptor) and RBV transporters but not IL10Rβ (Type III IFN-λ receptor). ER stress markers (BiP, IRE1α and peIF2α) and autophagy response (LC3II, Beclin 1 and ATG5) were induced in HCV infected chronic liver disease (CLD) and LC patients. Liver biopsies (CLD) show a 50% reduced expression of IFNAR1 and RBV transporters. Furthermore, the expression of IFNAR1 and RBV transporters was impaired in almost all LC patients.

**Conclusion:**

HCV infection induces ER stress and autophagy response in infected PHH and chronically infected liver tissues. The expression of IFNAR1, IFNγR1 and RBV transporters were significantly impaired in CLD and cirrhotic livers. Our study provides a potential explanation for the reduced response rate of IFN-α and RBV combination therapy in HCV infected patients with liver cirrhosis.

## Introduction

Hepatitis C virus (HCV) infection affects 150–200 million individuals worldwide [1], [2] and leads to a high rate of chronic liver diseases (CLD), which often progresses to liver cirrhosis (LC) and hepatocellular carcinoma (HCC). This infection is the leading cause of liver transplantation in the United States [3]. Antiviral therapy that clears HCV infection has improved patient quality of life and has reduced incidence of liver disease progression and HCC [4], [5]. Combination therapy of interferon (IFN)-α and ribavirin (RBV) with either a protease or polymerase inhibitor is now the prevalent treatment option for chronic HCV infection. Although this newly-approved therapy has improved HCV clearance rate, the treatment response has not improved among individuals who are initially non-responders to IFN-α and RBV [6], [7] therapy. However, this therapy is still the standard of care for HCV infection in many parts of the world with no access to newly-approved antiviral drugs. Viral and host factors have been implicated in the failure of IFN-α and RBV treatment for chronic HCV infection and disease progression [8]. We propose that a better understanding of IFN-α and RBV resistance mechanisms would help us improve viral clearance and reduce the risk of HCV-associated liver disease.

A recent review describes the viral factors that influence response to IFN-α and RBV treatment, including HCV genotype and the patient's baseline viral load [9]. Clinical studies have reported that a low baseline viral load is an independent predictive factor for sustained virological response (SVR) [8]. Age, gender, race, obesity, insulin resistance, pre-activation of IFN-inducible genes, degree of liver fibrosis, and IL-28B genetic polymorphisms are host-related factors associated with poor treatment performance [8]. Single nucleotide polymorphisms near the type III IFN-λ gene are strongly correlated with the level of treatment response [10]. Liver cirrhosis is another host-related factor affecting the success of IFN-α and RBV combination therapy of chronic HCV infection [11]–[13]. Although many clinical studies have confirmed the association of host and viral-related factors with viral clearance by IFN-α and RBV therapy, the molecular mechanisms of how these factors affect HCV treatment outcomes are not well understood.

Binding of IFN-α to type I IFN-receptors (IFNAR1 and IFNAR2) expressed on hepatocytes activates receptor-associated JAK kinases, leading to the phosphorylation of STAT1 and STAT2. This phosphorylated protein complex and IFN regulatory factor 9 enter the nucleus and initiate antiviral gene transcription. The activation of JAK-STAT signaling in infected hepatocytes is critical for IFN-α to induce antiviral clearance [14]. Another recent review described the modulation of HCV infection by JAK-STAT signaling via multiple mechanisms [15]. The importance of cellular JAK-STAT signaling in the IFN-α antiviral response has also been confirmed in our laboratory using stable sub-genomic HCV replicon cell lines, as well as an infectious HCV cell culture system [16]–[18]. We demonstrated that HCV replication in a replicon cell line expressing a truncated IFNAR1 remained resistant to IFN-α treatment. Overexpression of wild type IFNAR1 in the same resistant cell line restored IFN-sensitivity and induced HCV clearance [17]. Recently, we reported that HCV replication in persistently-infected Huh-7.5 cells induced endoplasmic reticulum (ER) stress and autophagy response and remains resistant to IFN-α and RBV combination treatment, whereas type III IFN-λ causes a strong and sustained antiviral response that leads to viral clearance [18]. Mechanisms of IFN-α and RBV resistance in HCV cell culture models were related to reduced expression of IFNAR1 and RBV transporters. The significance of these cell culture studies needs further confirmation using tissues from chronic HCV-induced liver diseases and an infected primary human hepatocyte-based cell culture model.

The primary aim of this study was to determine if increased ER stress and autophagy response are related to impaired expression of type I and type II IFN signaling in HCV-infected human liver tissue. Our results show ER stress and autophagy marker expression are induced in HCV-infected primary human hepatocytes and liver tissues of patients with CLD. Compared to normal human liver tissues, ER stress and autophagy response are increased during CLD, but IFNAR1 expression is significantly reduced in HCV-induced CLD with or without cirrhosis. These data verify the results of our previous cell culture studies indicating impaired type I IFN signaling during CLD and provide an explanation for the reduced viral clearance in cirrhotic patients receiving IFN-α and RBV combination therapy.

## Materials and Methods

### Reagents

A rabbit anti-human IFNAR1 (Abcam Inc., Cambridge, MA), a mouse anti-human IL-10Rβ (IFNλR, R&D Systems Inc., Minneapolis, MN), rabbit anti-human BiP, IRE1α, peIF2α, CHOP, Beclin 1, ATG5, LC3B, and GAPDH (Cell signaling Technology, Beverly, MA), IFNAR2, IFNγR1, CNT1 (Santa Cruz Biotechnology Inc., Santa Cruz, CA), HCV-core (Thermo Fisher Scientific, Waltham, MA), HCV-NS3 (Virogen, Watertown, MA), ENT1 (Abgent, San Diego, CA). A horseradish peroxidase-coupled antirabbit or antimouse IgG was used as secondary antibodies obtained from Cell Signaling Technology, Beverly, MA. Control siRNA (siIRR) and siRNAs against PERK and ATG7 (Life Technologies, Carlsbad, CA) were as described previously [18].

### Primary human hepatocytes (PHH) culture

Primary human hepatocytes obtained from a commercial source (XenoTech, LLC, Kansas City, MO) were cultured with a hepatocyte culture media with 10% human serum. After 24 hours of incubation in the CO_2_ incubator, cells were infected with the cell culture derived JFH-ΔV3-Rluc virus (HCV genotype 2a) with a MOI (multiplicity of infection) of 0.1. The chimera JFH-ΔV3-Rluc virus used in the development of HCV cell culture system in Huh-7.5 cells was obtained from Curt Hagedern laboratory [19]. After 18 hours of infection, hepatocytes were replaced with fresh hepatocyte culture media (XenoTech, LLC, Kansas City, MO) supplemented with 10% human serum (Invitrogen, Brown deer, WI). Uninfected or infected PHH were harvested every 2 days and cell pellets were used for RNA isolation and Western blot analysis.

### Ethics Statement

The study was conducted after IRB approval from the office of the senior Vice-President of Tulane University Health Sciences Center, New Orleans, LA, USA. The study was also received IRB approval from Ochsner Medical Center, New Orleans in order to collect explant liver tissues. All the patients were informed about the purpose of this study. Informed written consent was obtained from each patient.

### Patients and liver samples

A total of twelve liver biopsy specimens from HCV infected chronic liver disease patients (CLD) were included in this study collected from Tulane Medical Center, New Orleans, LA. A total of eighteen explant livers (nine HCV infected with liver cirrhosis and nine HCV negative, HBV negative explants liver with cirrhosis) were collected from Ochsner Medical Center, New Orleans, LA were also included in this study. A serum antibody to HCV was detected by an enzyme linked immunosorbent assay (ELISA) and a serum HCV RNA level was quantified by quantitative RT-qPCR assay. The liver tissues derived from HCV negative and HBV negative patients with a history of alcohol intake, Laennec's and cryptogenic cirrhosis as well as non-alcoholic steatohepatitis (NASH). These patients had no history of other viral infection (HCV, HBV or HIV). Nine normal liver tissues obtained from National Disease Research Interchange (NDRI), Philadelphia, PA were included as control.

### RT-qPCR for quantification of HCV RNA

The HCV-RNA level in infected human hepatocytes was determined using real-time RT-qPCR as described previously [20]. Briefly, one microgram of cellular RNA was used to amplify the 5′-untranslated region (UTR) of the HCV genome using sense (5′-TCTTCACGCAGAAAGCGTCTA-3′) and anti-sense (5′-CGGTTCCG CAGACCACTATG-3′) primers. The probe (5′-/56-FAM/TGAGTGTCG/ZEN/TG CAGCCTC CAGGA/3IBκFQ/-3′) labeled at the 5′ ends with a 6-carboxyfluorescein (FAM) fluorophore reporter molecule and ZEN-Iowa Black FQ (IBFQ) double quenchers were used to reduce the background and increase the signal (Integrated DNA Technologies Inc, Coralville, Iowa). The RT-qPCR assay was carried out in 20 µL containing 10 µL of iQ supermix (Bio-Rad Laboratories Inc., Hercules, CA), 0.25 µM of each primers, probe, and 4 µL of cDNA product obtained from the RT reaction. The amplification was carried out using a standard program (48°C for 30 minutes, 95°C for 10 minutes, followed by 44 additional cycles); each cycle includes a denaturation step at 95°C for 15 seconds, then an annealing and extension step at 60°C for 1 minute. Amplification, data acquisition, and analysis were carried out using a CFX96 Real-Time instrument using CFX manager software (Bio-Rad Laboratories Inc. Hercules, CA).

### Detection of HCV RNA by reverse transcription (RT)-nested PCR

Detection of HCV RNA in infected human hepatocytes was carried out by RT-nested PCR [21]. Briefly, cDNA synthesis was carried out using one microgram of total RNA along with outer antisense primer 5′- CACTACTCGGCTAGCAGT-3′ (0.25 µM) utilizing the same protocol for Real-Time PCR. Amplification of cDNA was performed in 50 µL reaction mixture containing 8 µL of 5× PCR buffer, 2.4 µL MgCl_2_ (25 mM), 2 U Taq DNA polymerase (Promega, Madison, WI), 0.25 µM outer sense primer (5′-CTGTGAGGAACTACTGTC-3′), and 10 µL of cDNA mixture as template. This was carried out for 40 cycles using the following program: denaturation for 30 sec at 95°C, annealing for 30 sec at 55°C, and extension for 1 min at 72°C. A second PCR was performed with a set of nested inner primers (sense: 5′-CACGCAGAAAGCGCCTAG-3′ and antisense: 5′- TTTATCCAAGAAAGGACCC-3′) by utilizing one-tenth volume of the first PCR product as a DNA template. The amplification cycles were identical to the first one. The nested-PCR products were migrated in a 1.5% agarose gel to visualize the amplification of positive strand HCV-RNA. Amplification of GAPDH mRNA served as an internal control.

### Western blot analysis

Western blotting was performed using our published protocol [18]. Infected primary hepatocytes were lysed in ice-cold RIPA buffer. Total protein content of the extract was quantified using a Bio-Rad protein assay kit (Bio-Rad, Hercules, CA). Hepatocytes were isolated from liver biopsies and lysed using an RIPA buffer described in a previous study [22]. Explant liver tissues were immediately collected in the RNase/DNase-free tubes containing Williams' media with 10% (v/v) FBS (Life Technologies, Carlsbad, CA). For the tissue samples, total liver protein was obtained by homogenizing liver tissue in 1 mL tissue grinders containing 1× PBS, 1% NP-40, 0.5% (v/v) Deoxycholate, 0.1% (v/v) SDS, 50 µg/mL PMSF, 5 µg/mL Aprotinin, 5 µg/mL Leupeptin, 1 µg/mL Pepstatin, and PhoSTOP phosphatase inhibitor (Roche Diagnostics Corporation, Indianapolis, IN). The sonicated protein lysates were then clarified by centrifugation at 12,000 rpm for ten minutes at 4°C. The clear supernatant was then transferred to new tubes, quantified, and stored at −20°C until used. An equal amount of protein from each sample was mixed in SDS-loading buffer, separated using NuPAGE (4–12%) gels and then transferred onto a nitrocellulose membrane (GE Healthcare, Buckinghamshire, UK). The membrane was soaked with a blocking solution for 2 h (at room temperature) and incubated with the diluted primary antibody (according to the manufacturer's instructions) at 4°C overnight. The next day, the membrane was washed and incubated with HRP-conjugated secondary antibody. The ECL detection reagent (GE Healthcare Life Sciences, Piscataway, NJ) was then added to the membrane according to the manufacturer's instructions. The membrane was then exposed to chemiluminescence film (GE Healthcare Life Sciences, Piscataway, NJ).

### Statistical analysis

All results were expressed as mean ± SD (standard deviation). Comparison between two groups was performed with a Student's t-test. To compare means within groups we performed one factor analysis of variance (ANOVA) using the GraphPad Prism software. We assumed that all measurements have normal probability distributions, which is expected for these types of data. P-value for statistical analysis was significant when p<0.05.

## Results

### HCV infection of PHH-induced ER stress, autophagy response, and downregulated IFNAR1 and IFNγR1 receptors and RBV transporters

**Figure 1:**
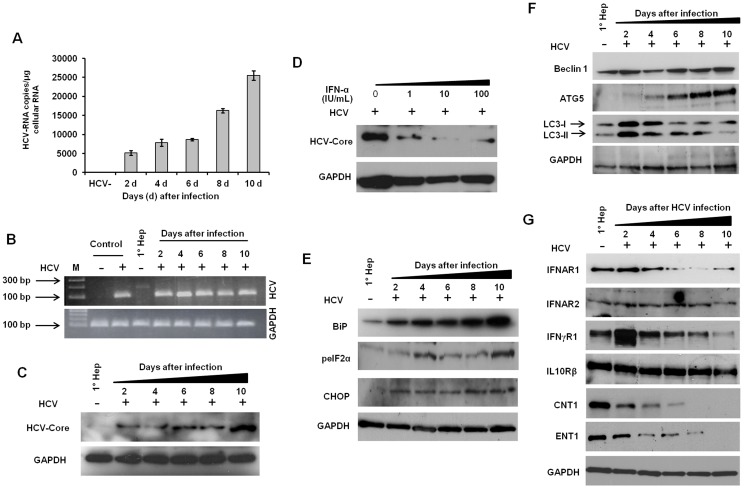
HCV replication in primary human hepatocytes (PHH) culture induces ER-stress and autophagy response and downregulates type I and type II IFN receptors and RBV transporters. Primary human hepatocytes were infected with cell culture derived JFH-ΔV3-Rluc HCV at a MOI = 0.1 for 18 hours. The infected cells were cultured in growth media supplemented with 10% (v/v) human serum. (**A**) HCV-RNA level measured by real-time RT-qPCR at different indicated time points. Uninfected PHH (HCV−) served as a negative control. (**B**) Ethidium bromide stained agarose gel electrophoresis picture showing the positive strand HCV-RNA was detected by RT-nested PCR. RNA from Huh-7.5 cells and *in vitro* transcribed HCV-RNA were used as negative (−) and positive (+) controls respectively. M: DNA ladder; 1° Hep: Uninfected primary human hepatocytes. (**C**) HCV-core protein level in infected primary hepatocytes was detected by Western blotting. GAPDH was used as a control. (**D**) IFN-α inhibits HCV core protein level in infected PHH. PHH were infected with JFH-ΔV3-Rluc virus for 18 hours, and after infection, cells were washed with PBS and cultured with hepatocyte culture media supplemented with 10% (v/v) human serum. After three days of infection, cells were treated with indicated concentrations of IFN-α for an additional three days. Finally, cells were harvested, lysed in RIPA buffer and HCV-core protein level was measured by Western blotting. (**E**) HCV infection induces ER stress. The level of ER-stress markers (BiP, peIF2α and CHOP) were induced in the PHH due to HCV infection. (**F**) Western blot shows expression of autophagy-related proteins (Beclin 1, ATG5 and LC3-II) in the infected hepatocytes. (**G**) The level of IFN receptors and RBV transporters were altered with HCV replication. The receptor expression of IFNAR1, IFNγR1, CNT1 and ENT1 gradually decreased with increasing time of infection. GAPDH was used as an internal control. 1° Hep: Uninfected primary human hepatocytes.

We initiated this study to verify ER stress and autophagy response seen in HCV-infected Huh-7.5 cells were also induced in primary human hepatocytes after HCV infection [18]. Prior studies reported that replication of HCV in a PHH is not as high when compared to the infected Huh-7.5 cells [23], [24]. A recent report showed HCV replication in PHH is induced 1000-fold when cultured in the presence of human serum instead of fetal bovine serum. The authors observed PHH cultured with human serum showed increased expression of hepatocyte differentiation markers [25], which encouraged us to establish a replication model in PHH by using cell-cultured HCV [18]. Infected PHH were collected at 0, 2, 4, 6, 8 and 10 days and examined to see if they supported HCV replication after infection. Initially, we measured Renilla luciferase activity of the protein lysate to quantify HCV replication in infected PHH, but the assay was not sensitive enough to detect luciferase signals above background levels. This result also indicated HCV replication may be low in infected PHH. Therefore, a more sensitive real-time RT-qPCR method was used to quantify HCV positive-strand RNA in PHH nucleic acid extracts, which was sensitive enough to detect HCV replication in PHH. We also observed HCV replication was significantly higher (P<0.001) when cells were cultured with human serum compared to cells cultured with fetal bovine serum (**Figure S1**). Using real-time RT-qPCR assays, we found the HCV RNA level in the infected human hepatocytes gradually increased to 2.5×10^4^ copies/1 µg total RNA from 0 to 10 days post-infection (**Figure 1A**). Viral titers of the infected PHH were comparable with HCV RNA levels in infected human liver [26]. Specificity of the RT-nested PCR amplified products was visualized with ethidium bromide staining of agarose gels following amplicon electrophoresis (**Figure 1B**). Viral core protein expression in the infected hepatocytes from 0 to 10 days was measured by Western blot analysis (**Figure 1C**). To confirm that a known antiviral molecule inhibits HCV replication in the infected PHH, one set of infected PHH culture was treated with IFN-α for 72 hours, and core protein was measured by Western blotting (**Figure 1D**). The increase in viral RNA levels and core protein expression over 10 days indicated that PHH cultured with human serum promoted HCV replication.

Next, protein extracts from infected human hepatocytes were used to measure expression levels of selected ER stress indicators, autophagy markers, IFN receptors, and RBV transporters by Western blotting. Expression levels of ER stress indicators, including the binding immunoglobulin protein (BiP), phosphorylation of eIF2α (peIF2α) and the C/EBP homology protein (CHOP), gradually increased throughout the duration of HCV infection (**Figure 1E**). We found the autophagy response in infected human hepatocytes was increased, and levels of Beclin 1, autophagy protein 5 (ATG5), and microtubule-associated protein 1A/1B-light chain 3 (LC3-II) correlated with the course of HCV infection (**Figure 1F**). We then examined if increased ER stress and autophagy response in the infected human hepatocyte model also correlated with decreased expression of IFNAR1. Expression levels of IFNAR1 (type I IFN) and IFNγR1 (type II IFN) receptors and RBV transporters (CNT1 and ENT1) in this model gradually decreased after HCV infection. Interestingly, levels of IFNAR2 (type I IFN receptor) and IL10Rβ (type III IFN receptor) were unaffected (**Figure 1G**). These Western blot results supported our cell culture studies of Huh-7.5 cells persistently infected with HCV [18]. To exclude the possibility that long-term culture of PHH *in vitro* could modulate the expression of IFNAR1, IFNAγR1, and RBV transporters, their expression was measured using uninfected primary human hepatocytes cultured at different time points. The expression levels of IFNAR1, IFNγR1, and RBV transporters did not change due to long-term culture of PHH (**Figure S2**). To confirm the association between induced ER stress and autophagy response with impaired expression of IFNAR1 and RBV transporters, one of the ER stress sensors (PERK) and autophagy gene (ATG7) were silenced by siRNAs in persistently HCV-infected Huh-7.5 cells. The expression level of IFNAR1 and RBV transporters (CNT1 and ENT1) were rescued after silencing PERK and ATG7 genes with siRNAs (**Figure S6**).

### Liver biopsies from chronic HCV patients show increased ER stress and autophagy response and decreased expression of IFNAR1, IFNγR1, and RBV transporters

**Figure 2:**
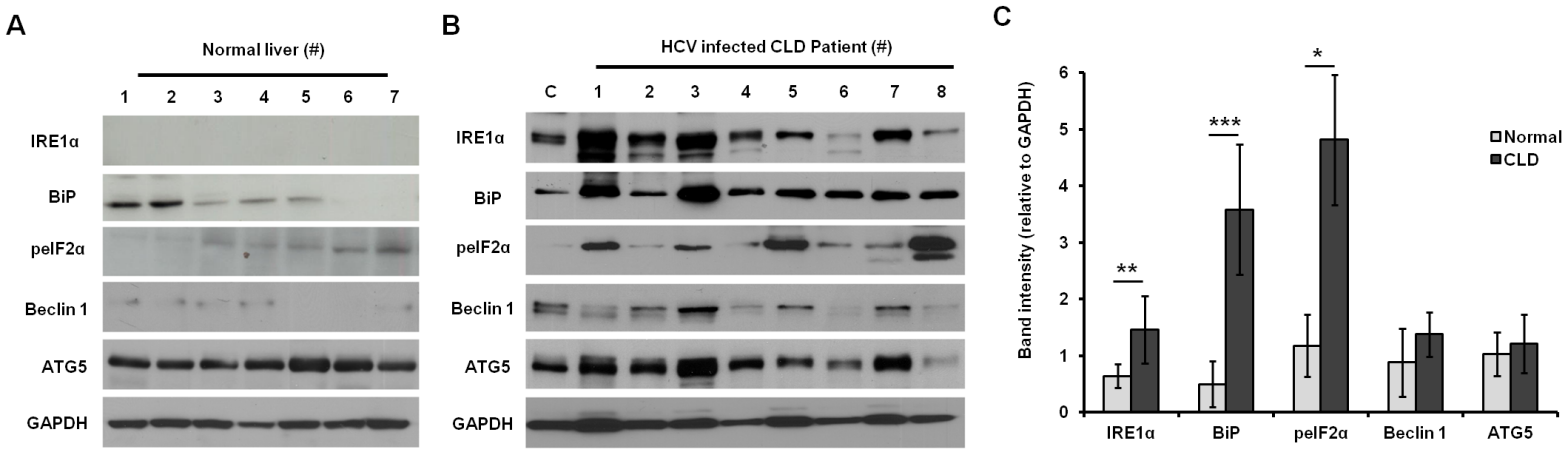
ER stress and autophagy response in HCV-infected liver biopsies from CLD patients. ER stress-related proteins (IRE1α, BiP, and peIF2α) and autophagy-related proteins (Beclin 1 and ATG5) were measured in the lysates prepared from liver biopsies by Western blotting (**A**) normal liver, and (**B**) HCV-infected CLD patients. (**C**) The relative band intensities of Western blot of HCV-infected CLD patients and normal liver samples were compared side-by-side by using Image J software. The relative values were reported as mean ± SD. *P<0.01, **P<0.0001, ***P<0.0001. C: Uninfected PHH.

Combination therapy of IFN-α and RBV produced a sustained antiviral response in 40–50% of patients infected with the HCV genotype 1 [27], [28]. These findings have been reproduced in many clinical studies, but the nature of this association has not been established. We tested if ER stress and autophagy response could also affect surface expression of IFNAR1 by examining liver biopsies from patients chronically infected with HCV. Hepatocytes were isolated from pretreatment liver biopsy samples using a standard protocol described previously [29]. Liver tissues from nine normal donor livers with no viral infection were used as controls. The clinical parameters of the HCV-infected patients and their serum viral titers are presented in **Figure S3**. Protein extracts of hepatocytes isolated from liver biopsies and normal liver tissues were examined for ER stress markers by Western blot analysis. Replication of HCV takes place in the ER-associated membranes and may induce an ER stress response due to increased accumulation of viral protein and sustained viral replication. This stress interferes with ER function. In this study, the ER stress marker expression was measured using protein extracts from liver biopsy hepatocytes using antibodies against inositol requiring 1 alpha (IRE1α), BiP, and peIF2α. Compared to normal donor liver tissues (**Figure 2A**), expression levels of ER stress markers IRE1α, BiP and peIF2α in HCV-infected CLD patients were significantly increased (^**^P<0.001, ^***^P<0.0001, and, ^*^P<0.01 respectively) (**Figure 2B and 2C**). Western blot results were quantified by measuring band intensities, which indicated ER stress response was significantly higher in CLD patients (**Figure 2C**). Levels of GAPDH were the similar in all samples, indicating the observed differences were not due to unequal amounts of proteins in the extracts.

**Figure 3:**
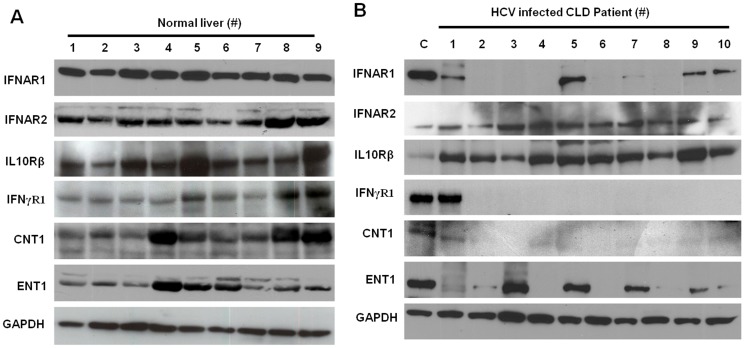
Expression of IFN receptors and RBV transporters in normal uninfected liver and HCV-infected liver samples. The levels of different type I (IFNAR1 and IFNAR2), type II (IFNγR1) and type III (IL10Rβ) IFN receptors and RBV transporters (CNT1 and ENT1) were measured by Western blotting using equal amounts of protein lysates from normal liver and biopsy specimens. (**A**) Nine normal liver tissues with no history of viral infection (HIV, HBV or HCV) were used as control. (**B**) Liver biopsies from ten HCV-infected CLD patients were examined. GAPDH was used as an internal control. C: Uninfected PHH.

Induction of autophagy response secondary to ER stress has been seen in liver disease with accompanying chronic HCV infection, steatohepatitis, alcoholic liver disease, and HCC. To determine if autophagy was induced or impaired in CLD and LC patients, we investigated hallmark autophagy genes such as Beclin 1 and ATG5 and found their protein levels were induced in HCV-infected CLD patients compared to controls (**Figure 2B and 2C**). We were unable to test all ER stress and autophagy markers due to a limited amount of protein extract available from the liver biopsies. Because ER stress and autophagy markers are induced in chronic HCV patients, we also measured expression levels of IFNAR1 and RBV transporters (ENT1 and CNT1) by Western blotting and compared normal liver and CLD samples (**Figure 3A**). The expression of IFNAR1 was detectable in 40% of chronic HCV patients by Western blot analysis (**Figure 3B**), and expression of ENT1, the major transporter for RBV uptake, was detectable in 50% of chronic HCV patients (**Figure 3B**). Interestingly, the expression of the type II IFN-γ receptor-chain 1 (IFNγR1) was also impaired in chronically-infected livers. The expression level of IL10Rβ (type III IFN) was detectable in all of the infected livers (**Figure 3B**). Expression levels of IFNAR1, IFNγR1 and RBV transporters were detectable in all normal uninfected liver samples, and GAPDH levels were comparable in all samples, indicating variations were not due to differences in the amount of proteins used in our assays.

### Lack of expression of IFNAR1 and RBV transporter in the explant liver tissues from patients with LC

**Figure 4:**
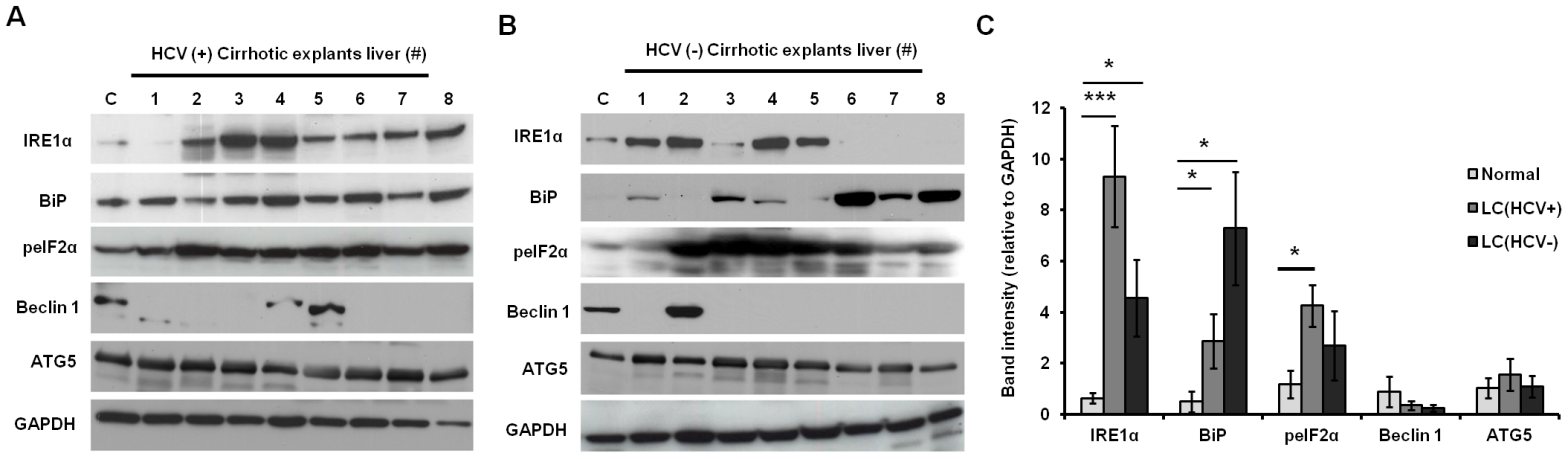
ER stress and autophagy response in LC patients (explant liver tissues) with or without the etiology of HCV infection. The ER stress-related proteins (IRE1α, BiP, and peIF2α) and autophagy-related proteins (Beclin 1 and ATG5) were detected by Western blot in (**A**) HCV infected (HCV+) LC patients, and (**B**) HCV uninfected (HCV−) LC patients. (**C**) The relative band intensity of each marker between normal and HCV-infected and –uninfected was compared using Image J software. The relative values were reported as mean ± SD and *P<0.01, ***P<0.0001. C: uninfected PHH.

**Figure 5:**
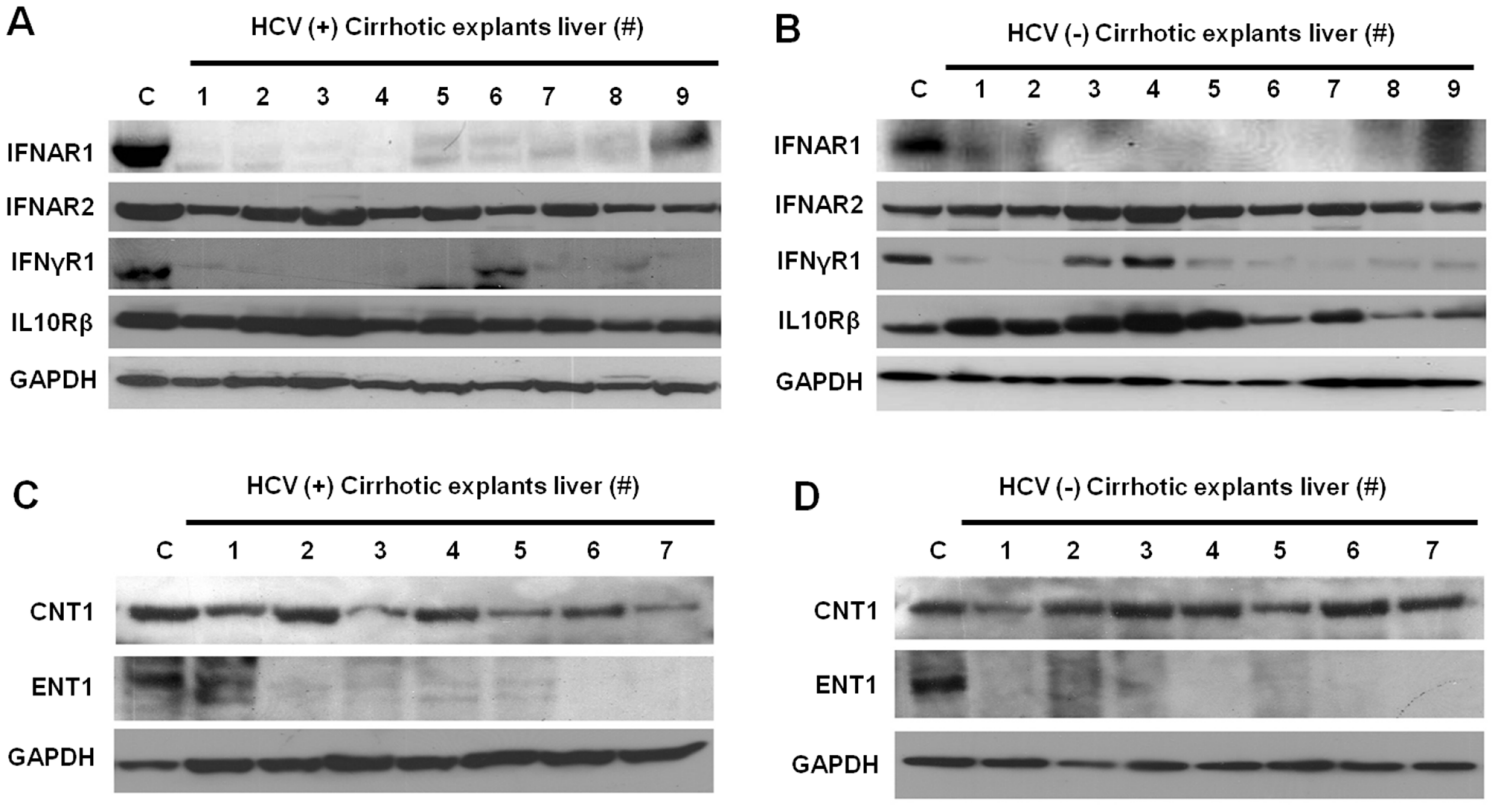
Expression of IFN receptors and RBV transporters in explant livers with cirrhosis. Equal amounts of protein lysates prepared from explant liver tissues and separated in 4–12% NuPAGE gels and the protein levels of different IFN receptors and RBV transporters were measured by Western blotting. Expression of type I, type II and Type III IFN-receptors were presented in (**A**) HCV infected (HCV+) explant livers and (**B**) HCV uninfected (HCV−) explant livers. Expression of RBV transporters presented in (**C**) HCV infected (HCV+) explant livers and (**D**) HCV uninfected (HCV−) explant livers. We were unable to measure RBV transporters in all samples. C: Uninfected PHH.

Liver cirrhosis is an end-stage disease with both viral and non-viral causes. Without successful antiviral therapy, the majority of liver cirrhotic patients will die due to liver- related complications, such as HCC and liver failure [5]. Standard IFN-α and RBV therapy induces a sustained antiviral response from only 25% of HCV genotype 1-infected patients with LC [29]. The reason for the low treatment response to this HCV therapy in cirrhotic patients is unknown. To verify the contribution of ER stress- and autophagy-related impaired expression of IFNAR1 and RBV transporters, we measured their expression levels using nine explant livers with HCV infection and nine explant livers without HCV infection. All explant livers used in our study were cirrhotic, as confirmed by histological examination by a pathologist. The demographic characteristics of patients with or without HCV are shown in **Figure S4**. Liver tissue from nine normal donor livers with no viral infection was used as a control. The expression of ER stress and autophagy markers was examined by subjecting protein extracts to Western blot analysis. The ER stress markers (BiP, IRE1α, and peIF2α) were induced in LC patients with or without HCV infection (**Figure 4A and 4B**). Autophagy marker Beclin1 was detected in two HCV-infected LC patients (25%, 2/8) and one uninfected LC patient (12%, 1/8). The level of ATG5 was unchanged in both HCV-infected and uninfected LC patients. Autophagy markers were downregulated in explant livers as compared to the expression levels of ER stress markers (**Figure 4C**). We then tested the expression levels of IFN receptors and RBV transporters in the same extracts using Western blotting. Surprisingly, IFNAR1 and IFNγR1 expression was undetectable in the majority of cirrhotic livers with or without HCV infection (**Figure 5A and 5B**). The expression of ENT1 was impaired in all cirrhotic livers with or without HCV infection (**Figure 5C and 5D**). All non-HCV cirrhotic livers showed undetectable IFNAR1 (100%) and ENT1 (100%) expression by Western blot analysis, and the IFNγR1 expression band was detectable in only two out of nine tissues (22%). These results suggest the expression of IFNAR1 and RBV transporters was severely impaired in patients with LC. However, the negative results of the Western blots are not due to low amounts of protein in the tissue extracts, as we saw stable expression of GAPDH levels in all samples.

**Figure 6:**
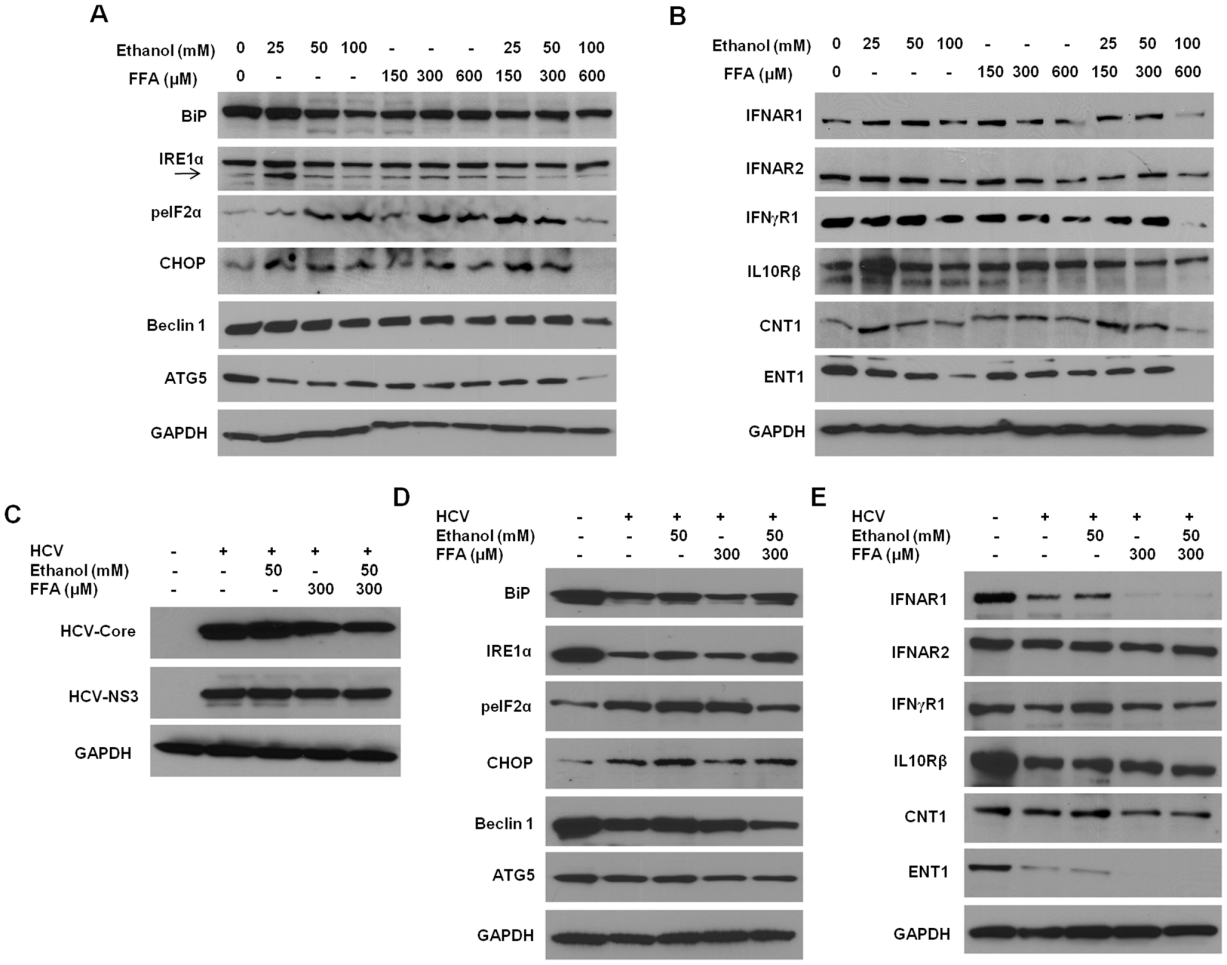
Ethanol and free fatty acid (FFA) induces ER stress. Huh-7.5 cells were treated with indicated concentrations of ethanol and FFA for 24 h. (**A**) ER stress-related proteins (BiP, IRE1α, peIF2α, and CHOP) and autophagy-related proteins (Beclin 1 and ATG5) were detected by Western blotting. (**B**) The expression level of IFN-receptors and RBV transporters was measured in ethanol and FFA treated cells. The effect on ER stress, autophagy response, the level IFN-receptors and RBV transporters in ethanol and FFA treated HCV-infected culture was also studied. (**C**) Persistently HCV infected Huh-7.5 cells were treated with indicated concentrations of ethanol and FFA for 24 h. HCV-core and HCV-NS3 protein levels were detected by Western blotting. (**D**) ER stress and autophagy-related proteins were detected by Western blotting. (**E**) The expression level of IFN-receptors and RBV transporters in ethanol and FFA treated HCV-infected culture was also measured by Western blotting. GAPDH was used as an internal control in all of the panels.

The activity of ER stress in cirrhotic livers was higher than that of autophagy markers. The downregulation of IFNAR1 without significant autophagy suggests IFNAR1 downregulation could occur due to ER stress. The ER stress inducer downregulates IFNAR1 expression [18], [30]. Because IFNAR1 expression was severely impaired compared to chronically-infected liver tissues, the mRNA level of IFNAR1 was examined using RT-PCR followed by Southern blotting. Interestingly, full-length mRNA of IFNAR1 was not detected in most of the cirrhotic livers (**Figure S5**). The primers and probes used to amplify full-length IFNAR1 are shown in **Table S1**. Liver cirrhosis also occurs due to chronic alcoholic and non-alcoholic liver disease. The non-HCV explant cirrhotic liver tissues used in our study were diagnosed as alcoholic liver disease and non-alcoholic steatohepatitis. The ER stress and autophagy response plays a role in viral- and non-viral-induced CLDs [31]–[38]. To understand why reduced expression of IFNAR1 occurs in non-viral liver diseases, we investigated the effect of ethanol and free fatty acids (FFA) alone or in combination on the expression levels of IFN receptors and RBV transporter proteins in Huh-7.5 cells. The induction of ER stress and autophagy markers were upregulated in Huh7.5 cell cultures treated with ethanol alone, FFA alone, and the combination ethanol plus FFA (**Figure 6**). Protein levels of peIF2α and CHOP were induced in Huh-7.5 cells by ethanol or FFA treatment, indicating induction of ER stress (**Figure 6A**). Autophagy response was not induced significantly after 24 hours of ethanol treatment, as protein levels of both Beclin 1 and ATG5 did not increase with higher concentrations of ethanol or FFA (**Figure 6A**). Interestingly, expression of IFNAR1, IFNAR2 (type I IFN receptor), and IFNγR1 (type II IFN receptor) was decreased with higher concentrations of ethanol or FFA and in a combination-treated culture. Similarly, expression levels of RBV transporters (both ENT1 and CNT1) were decreased with higher concentration of ethanol or FFA or when treated in combination (**Figure 6B**). We also measured the induction of ER stress, autophagy response, and levels of IFN receptors in HCV-infected cultures treated with ethanol and FFA (**Figures 6C–E**). The presence of HCV infection was confirmed by detecting HCV core and NS3 protein expression by Western blotting (**Figure 6C**). Interestingly, the protein levels of peIF2α and CHOP were induced when alcohol and FFA were added to the HCV-infected culture (**Figure 6D**). In addition, expression levels of IFNAR1 and the RBV transporter ENT1 were severely impaired in HCV-infected cells treated with ethanol and FFA (**Figure 6E**).

## Discussion

In this study, we validated the results of previous HCV cell culture findings by confirming ER stress and autophagy responses are induced in chronic viral infection of human hepatocytes, leading to impaired expression of IFNAR1, IFNγR1, and RBV transporters. We also established an HCV replication model using PHH and found HCV replication in PHH can be inhibited by IFN-α. Using this system, we verified HCV replication in PHH induced ER stress and autophagy response but impaired expression of IFNAR1, IFNγR1, and RBV transporters. Expression of the IFN-λ receptor was not affected by ER stress and autophagy response.

The ER stress- and autophagy-mediated degradation of IFNAR1 and RBV transporter expression was verified using liver biopsy samples from chronic HCV patients. We show ER stress and autophagy markers are induced in chronic HCV-infected liver tissues. Our results confirm other related studies [33]–[35]. For example, Asselah et al. [33] observed abnormally-dilated ER, indicative of ER stress, due to viral protein accumulation in liver biopsies of patients with chronic HCV and Hepatitis B infection. Ait-Goughoulte et al. [34] showed HCV genotype 1a infection of immortalized primary human hepatocytes induced autophagy response, expression of ATG5, and LC3 accumulation. Shuda et al. [35] examined the expression of ER stress markers (ATF6, XBP1, and GRP78) using 13 surgically-resected human HCC liver tissues and normal liver tissues by Northern blot analysis. They observed increased levels of ER stress markers in HCV infection and HCC samples. The expression of IFNAR1 and RBV transporters was absent in 40–50% of chronic HCV patients, which correlates with clinical studies explaining why only 50% of chronic HCV patients respond to peginterferon and RBV combination therapy. Interferon-γ is not an effective treatment of chronic HCV patients who are non-responders to IFN-α [39], but the reason is unknown. Impaired expression of IFNγR1 in chronic HCV infection may provide an explanation why IFN-γ therapy alone is not effective in chronic HCV patients. The results from liver biopsy assays are also consistent with our previous cell culture study, indicating chronic ER stress and autophagy response is associated with impaired expression of IFNAR1 and RBV transporters.

The sustained viral response of IFN-α and RBV combination therapy only occurs in 25% of chronic HCV patients with LC, which could be the result of several viral- and host-related factors [40]. Evidence for viral factor contribution is limited because viral strains with mutations causing IFN-α and RBV resistance has not been developed. The mechanism of IFN-α and RBV resistance is likely connected to host-related factors, which is supported by numerous studies [40]. Liver cirrhosis in chronic HCV infection is an independent predictor of poor response to IFN-α and RBV therapy [40], but there is no explanation why cirrhotic patients show less viral clearance with IFN-α and RBV combination therapy. We investigated the expression of ER stress indicators, autophagy markers, and IFNAR1 and RBV transporters using cirrhotic livers obtained during liver transplantation. The ER stress markers are upregulated in cirrhotic livers compared to autophagy markers, but the expression of IFNAR1 and RBV transporter ENT1 was severely impaired in the explant liver samples with or without HCV infection. Our results support the findings of other investigators who have shown ER stress and autophagy responses are induced in CLD of viral and non-viral etiology [37], [38].

Several studies have demonstrated autophagy in chronic HCV and CLD patients due to alcohol and FFA accumulation [37]. The lack of IFNAR1 and RBV transporter expression provides a potential explanation why cirrhotic patients are less responsive to the combination therapy of IFN-α and RBV. These results verify our hypothesis that IFNAR1 expression is impaired due to ER stress and autophagy responses in HCV cell culture, as well as in CLD and LC tissues, although autophagy responses are much lower in during LC compared to CLD. The reasons for IFNAR1 and RBV transporter degradation, even during impaired autophagy, are unknown. We examined the stability of IFNAR1 mRNA in explant livers by RT-PCR, followed by Southern blotting. Full-length mRNA for IFNAR1 was not detected in the explant livers. The IRE1α RNase activity due to ER stress was also implicated in the degradation of several cellular mRNAs, including proinsulin mRNA [41]. In addition, IRE1α may be an endonuclease that plays a role in regulating the decay of numerous cellular mRNA, including IFNAR1, in response to cellular ER stress as a cell survival defense mechanism. The contribution of IRE1α-mediated gene expression in the cirrhotic liver should be examined in the future. In our study, we observed Beclin 1 was induced in HCV-infected CLD patients (**Figure 2B and C**) but was undetectable in most of the cirrhotic explant livers with or without HCV infection (**Figure 4**). However ATG5, a well-known marker for macroautophagy, is expressed in all cirrhotic livers, which indicates autophagy in the cirrhotic liver may be Beclin 1-independent. This possibility is supported by a recent study showing the autophagy process can be induced without Beclin 1 [42]. However, we observed ER stress markers are induced more in cirrhotic livers. A recent review article discusses various stress-responsive transcription factors that regulate autophagy responses in mammalian cells [43]. We suspect stress-responsive transcription factors, induced secondarily to ER stress, could also regulate autophagy processes that downregulate IFNAR1 and RBV transporter expression in cirrhotic livers.

In conclusion, we present evidence that HCV-induced ER stress and autophagy degrades IFNAR1, IFNγR1, and RBV transporters by using an HCV cell culture model, primary human hepatocytes, and HCV-infected livers. However, the significance of reduced expression of type I and type II IFN receptors in CLD and LC is not clear. Chronic liver disease, whether due to viral or non-viral causes, reduces expression of IFNAR1 and suggests suppression of host innate immunity and liver disease progression. Suppression of host immunity favors carcinogenesis, which may be why LC is a potential risk factor for HCC development. Interestingly, expression of the IFN-λ receptor is not affected by ER stress and autophagy mechanisms influenced by HCV. We propose IFN-λ treatment can be used to circumvent the IFN-α and RBV resistance mechanisms of HCV infection. Understanding how ER stress and autophagy responses play a role in chronic HCV infection and IFN resistance could help reduce HCV-associated liver diseases in humans.

## Supporting Information

Figure S1 **Real-time RT-PCR showing HCV-RNA levels in PHH culture in presence of human serum (HS+) and fetal bovine serum (HS−).** Cells were infected with JFH-ΔV3-Rluc virus (MOI = 0.1) for 18 hours. The infected cells were washed with PBS and cultured with hepatocyte culture media supplemented with either 10% (v/v) human serum (HS+) or 10% (v/v) fetal bovine serum (HS−). HCV replication was measured by real-time RT-qPCR at different indicated time points. HCV replication was compared in the presence (HS+) or absence (HS−) of human serum. **P<0.001, ***P<0.0001.
(TIF)

Figure S2 **The expression of different IFN receptors and RBV transporters did not change due to the long-term PHH culture.** Cells were cultured in hepatocyte culture media supplemented with 10% (v/v) human serum and the levels of different indicated proteins were measured at different indicated time points by Western blotting. GAPDH was used as an internal control.
(TIF)

Figure S3 **Characteristics of all 12 HCV-infected CLD patients.** Viral titer in serum was measured by RT-qPCR to amplify a specific portion of the 5′-untranslated region (5′-UTR) of the HCV genome. HCV genotype and sub-type were determined by direct sequencing. The amplified nucleic acid was sequenced bi-directionally using dye-terminator chemistry. Results were obtained based on comparison with a database derived from GenBank sequences. Histopathology for fibrosis and steatosis were determined by the Pathologist from H&E staining of biopsy specimens. _#_c: primary human hepatocytes as control.
(TIF)

Figure S4 **Characteristics of HCV-infected LC patients.** HCV-RNA level in serum was measured by RT-qPCR. HCV genotype and sub-type were determined by direct sequencing. NR: non-responder, PR: partial-responder.
(TIF)

Figure S5 **RT-nested PCR amplification and Southern Blotting of IFNAR1 mRNA from explant livers.** Total RNA was isolated from the explant liver. The first round PCR amplification of the 3′ end of IFNAR1 was performed using the S/901 and AS/1926 primer set listed in **Table S1**. The second round, hemi-nested PCR amplification was conducted using the S/1345 and AS/1926 primer set listed in **Table S1**. The hemi-nested PCR products were then loaded onto a 2% agarose gel. The DNA was then transferred to a membrane (Bio-Rad, Hercules, CA). The membrane was then washed, air dried, and hybridized in hybridization solution (5× SSC, 20 mM NaH_2_PO_4_, 7%SDS, 10× Denhardt, salmon sperm DNA) for three hours at 50°C. Probe S/1633 (**Table S1**) was then added directly to the hybridization solution. The membrane and probe were then hybridized for 14 hours at 50°C. Following hybridization, the membrane was washed (3× SSC, 25 mM NaH_2_PO_4_, 5%SDS, 10× Denhardt, salmon sperm DNA) and finally exposed in XAR film (Kodak, Rochester, NY). The 5′ end of IFNAR1 was PCR amplified using the S/241 and AS/402 primer set and probes S/253 (**Table S1**) utilizing the method described above. (**A**) Schematic presentation for amplification of IFNAR1 gene. aa: amino acids; nt: nucleotides; S: sense primer; AS: anti-sense primer. RT-nested PCR amplification and Southern blotting of 5′ and 3′ region of IFNAR1 in (**B**) non-HCV (HCV−) LC patients, and (**C**) HCV infected (HCV+) LC patients. SB: Southern blotting.
(TIF)

Figure S6 **Inhibition of ER stress and autophagy response rescued IFNAR1 and RBV transporters.** Persistently HCV-infected Huh-7.5 cells were transfected with 100 pico-mole concentrations of control siRNA (siIRR) and siRNAs against PERK (ER stress sensor) and ATG7 (autophagy gene) for 72 h. The expression of IFN-α receptor 1 (IFNAR1) IFN-γ receptor 1 (IFNγR1) and RBV transporters (CNT1 and ENT1) was detected by Western blotting. GAPDH was used as an internal control.
(TIF)

Table S1 **Nucleotide sequences of primers and probes used to demonstrate IFNAR1 mRNA by RT-PCR and Southern blotting.**
(TIF)
