# Correction: Impaired Expression of Type I and Type II Interferon Receptors in HCV-Associated Chronic Liver Disease and Liver Cirrhosis

**DOI:** 10.1371/journal.pone.0114456

**Published:** 2014-11-20

**Authors:** The PLOS ONE Staff

The ninth author’s name is incorrect. The correct name is: Humberto E. Bohorquez.
