# Expression of Concern: Impaired expression of type I and type II interferon receptors in HCV-associated chronic liver disease and liver cirrhosis

**DOI:** 10.1371/journal.pone.0320610

**Published:** 2025-03-19

**Authors:** The PLOS One Editors

The corresponding author stated that the primary data underlying this article [1,2] are no longer available, and the primary data were not provided with the published article [1,2], contrary to the Data Availability statement.

This article [1] therefore does not comply with the PLOS Data Availability policy. In light of this, the *PLOS One* Editors issue this Expression of Concern.
