# Supplementary figures and images for: Impaired Expression of Type I and Type II Interferon Receptors in HCV-Associated Chronic Liver Disease and Liver Cirrhosis
Source: PLoS One. 2014 Sep 29;9(9):e108616. doi: 10.1371/journal.pone.0108616 (PMC4180933; doi:10.1371/journal.pone.0108616)

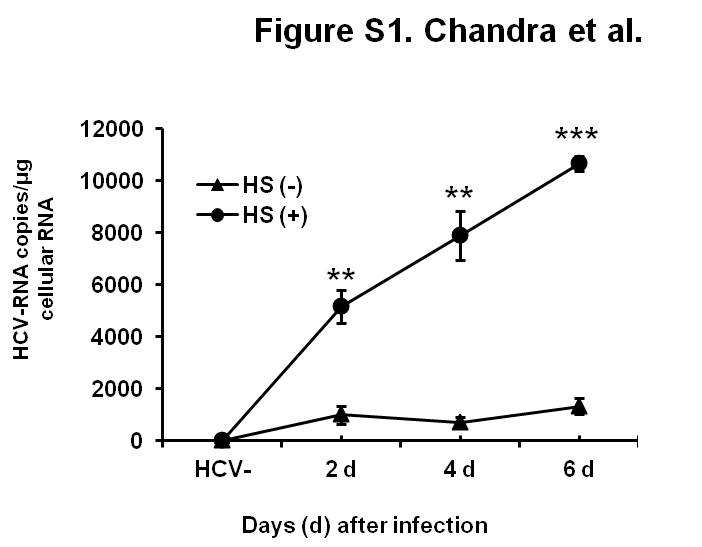

Supplement: Figure S1 — Real-time RT-PCR showing HCV-RNA levels in PHH culture in presence of human serum (HS+) and fetal bovine serum (HS−). Cells were infected with JFH-ΔV3-Rluc virus (MOI = 0.1) for 18 hours. The infected cells were washed with PBS and cultured with hepatocyte culture media supplemented with either 10% (v/v) human serum (HS+) or 10% (v/v) fetal bovine serum (HS−). HCV replication was measured by real-time RT-qPCR at different indicated time points. HCV replication was compared in the presence (HS+) or absence (HS−) of human serum. **P<0.001, ***P<0.0001. (TIF) [file pone.0108616.s001.tif]

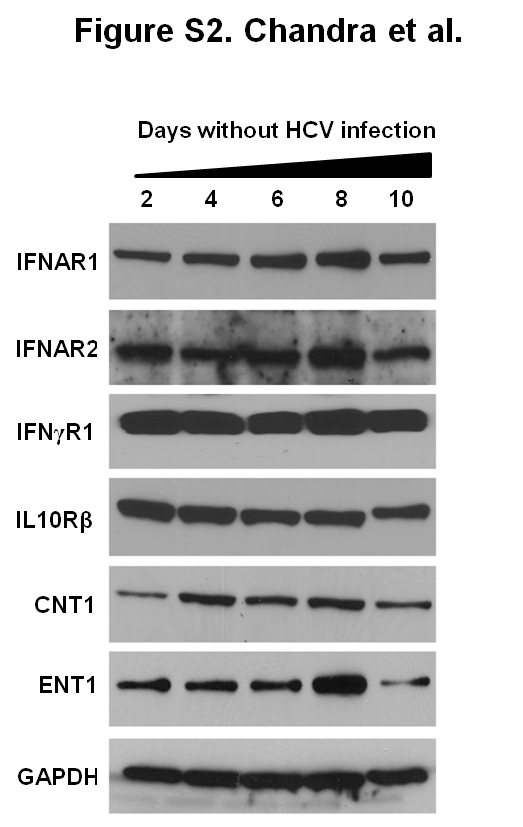

Supplement: Figure S2 — The expression of different IFN receptors and RBV transporters did not change due to the long-term PHH culture. Cells were cultured in hepatocyte culture media supplemented with 10% (v/v) human serum and the levels of different indicated proteins were measured at different indicated time points by Western blotting. GAPDH was used as an internal control. (TIF) [file pone.0108616.s002.tif]

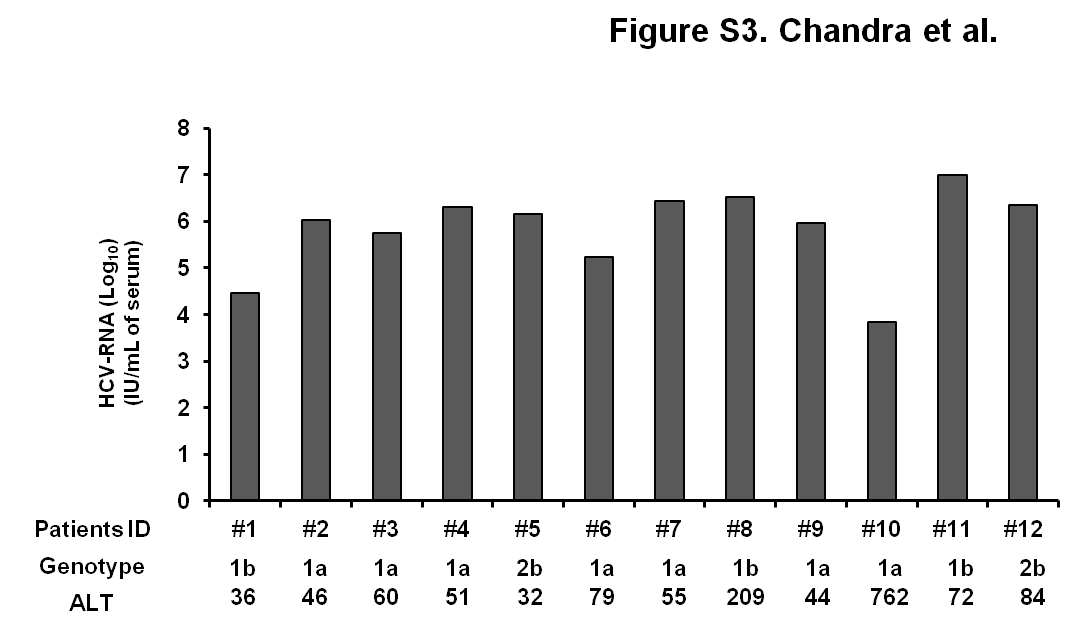

Supplement: Figure S3 — Characteristics of all 12 HCV-infected CLD patients. Viral titer in serum was measured by RT-qPCR to amplify a specific portion of the 5′-untranslated region (5′-UTR) of the HCV genome. HCV genotype and sub-type were determined by direct sequencing. The amplified nucleic acid was sequenced bi-directionally using dye-terminator chemistry. Results were obtained based on comparison with a database derived from GenBank sequences. Histopathology for fibrosis and steatosis were determined by the Pathologist from H&E staining of biopsy specimens. #c: primary human hepatocytes as control. (TIF) [file pone.0108616.s003.tif]

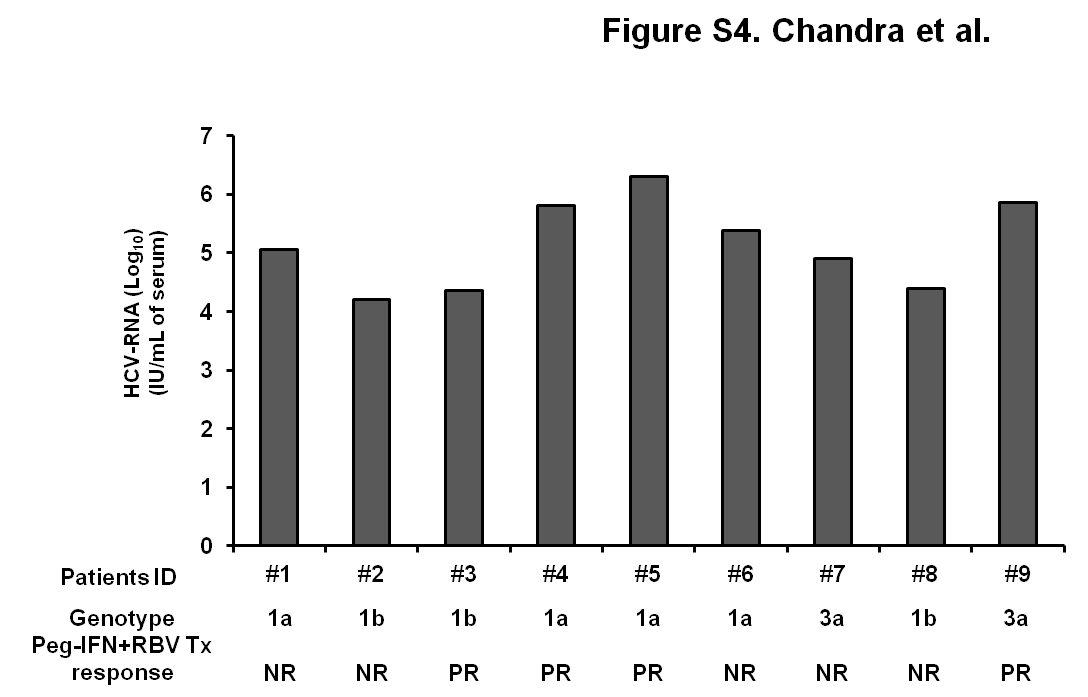

Supplement: Figure S4 — Characteristics of HCV-infected LC patients. HCV-RNA level in serum was measured by RT-qPCR. HCV genotype and sub-type were determined by direct sequencing. NR: non-responder, PR: partial-responder. (TIF) [file pone.0108616.s004.tif]

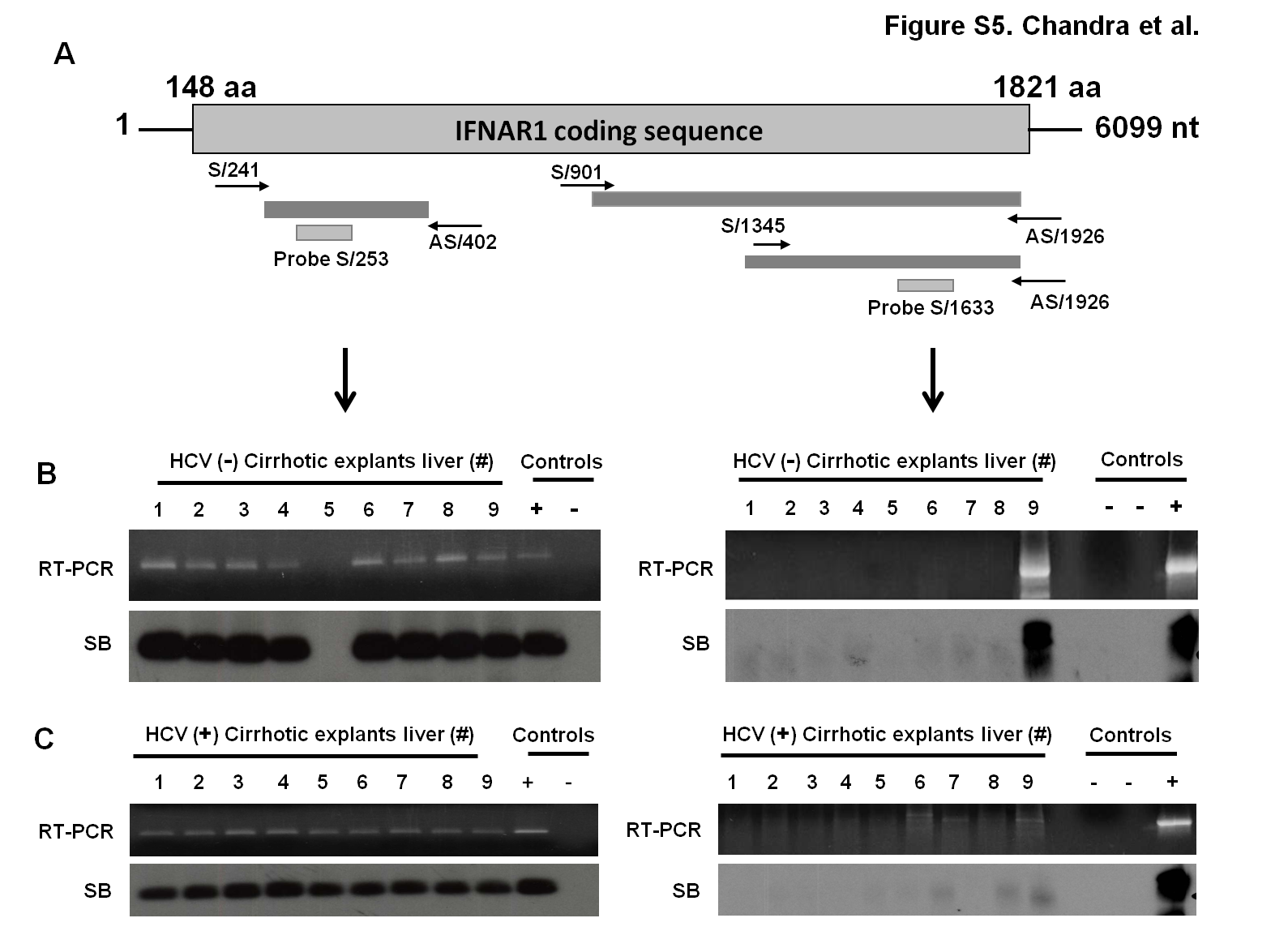

Supplement: Figure S5 — RT-nested PCR amplification and Southern Blotting of IFNAR1 mRNA from explant livers. Total RNA was isolated from the explant liver. The first round PCR amplification of the 3′ end of IFNAR1 was performed using the S/901 and AS/1926 primer set listed in Table S1. The second round, hemi-nested PCR amplification was conducted using the S/1345 and AS/1926 primer set listed in Table S1. The hemi-nested PCR products were then loaded onto a 2% agarose gel. The DNA was then transferred to a membrane (Bio-Rad, Hercules, CA). The membrane was then washed, air dried, and hybridized in hybridization solution (5× SSC, 20 mM NaH2PO4, 7%SDS, 10× Denhardt, salmon sperm DNA) for three hours at 50°C. Probe S/1633 (Table S1) was then added directly to the hybridization solution. The membrane and probe were then hybridized for 14 hours at 50°C. Following hybridization, the membrane was washed (3× SSC, 25 mM NaH2PO4, 5%SDS, 10× Denhardt, salmon sperm DNA) and finally exposed in XAR film (Kodak, Rochester, NY). The 5′ end of IFNAR1 was PCR amplified using the S/241 and AS/402 primer set and probes S/253 (Table S1) utilizing the method described above. (A) Schematic presentation for amplification of IFNAR1 gene. aa: amino acids; nt: nucleotides; S: sense primer; AS: anti-sense primer. RT-nested PCR amplification and Southern blotting of 5′ and 3′ region of IFNAR1 in (B) non-HCV (HCV−) LC patients, and (C) HCV infected (HCV+) LC patients. SB: Southern blotting. (TIF) [file pone.0108616.s005.tif]

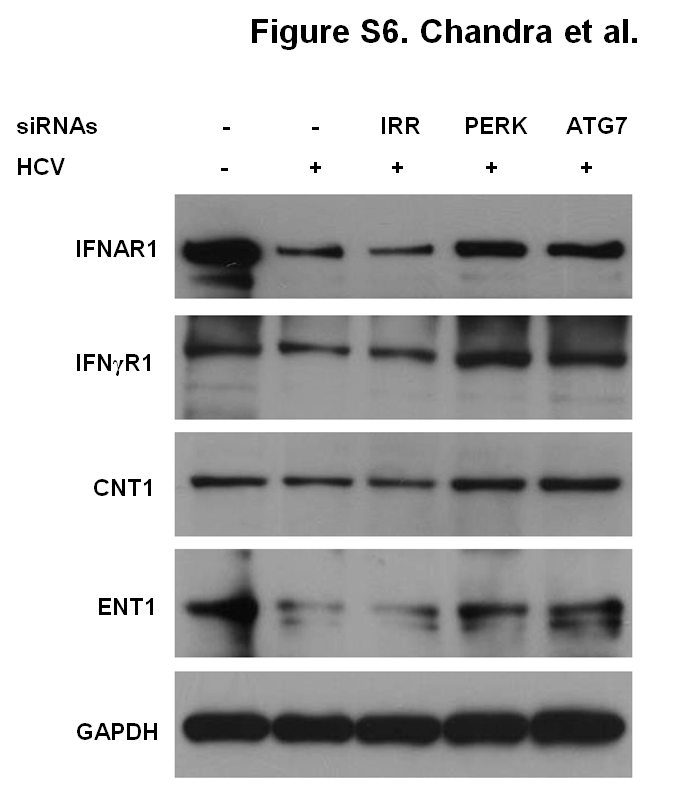

Supplement: Figure S6 — Inhibition of ER stress and autophagy response rescued IFNAR1 and RBV transporters. Persistently HCV-infected Huh-7.5 cells were transfected with 100 pico-mole concentrations of control siRNA (siIRR) and siRNAs against PERK (ER stress sensor) and ATG7 (autophagy gene) for 72 h. The expression of IFN-α receptor 1 (IFNAR1) IFN-γ receptor 1 (IFNγR1) and RBV transporters (CNT1 and ENT1) was detected by Western blotting. GAPDH was used as an internal control. (TIF) [file pone.0108616.s006.tif]

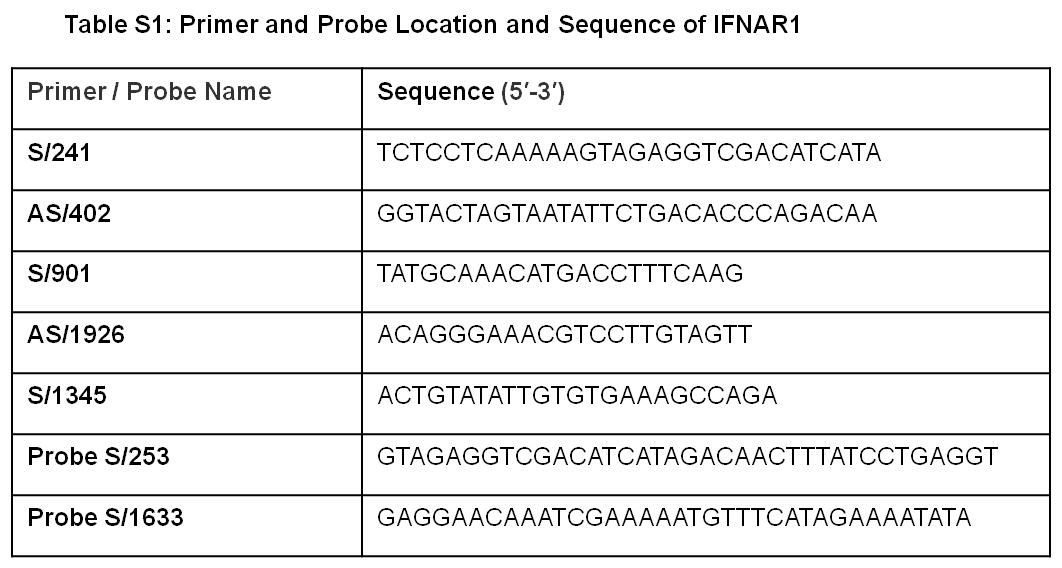

Supplement: Table S1 — Nucleotide sequences of primers and probes used to demonstrate IFNAR1 mRNA by RT-PCR and Southern blotting. (TIF) [file pone.0108616.s007.tif]
